# Turning adversity into opportunity: Small extracellular vesicles as nanocarriers for tumor‐associated macrophages re‐education

**DOI:** 10.1002/btm2.10349

**Published:** 2022-06-09

**Authors:** Dario Donoso‐Meneses, Aliosha I. Figueroa‐Valdés, Nicolás Georges, Hugo E. Tobar, Francisca Alcayaga‐Miranda

**Affiliations:** ^1^ Laboratory of Nano‐Regenerative Medicine, Centro de Investigación e Innovación Biomédica (CIIB), Faculty of Medicine Universidad de Los Andes Santiago Chile; ^2^ Consorcio Regenero Chilean Consortium for Regenerative Medicine Santiago Chile; ^3^ IMPACT Center of Interventional Medicine for Precision and Advanced Cellular Therapy Santiago Chile; ^4^ School of Medicine, Faculty of Medicine Universidad de Los Andes Santiago Chile; ^5^ Cells for Cells Santiago Chile

**Keywords:** cancer, immunotherapy, macrophages, nanocarrier engineering, small extracellular vesicles, TAMs re‐education

## Abstract

Currently, small extracellular vesicles (sEV) as a nanoscale drug delivery system, are undergoing biotechnological scaling and clinical validation. Nonetheless, preclinical pharmacokinetic studies revealed that sEV are predominantly uptaken by macrophages. Although this “sEV‐macrophage” propensity represents a disadvantage in terms of sEV targeting and their bioavailability as nanocarriers, it also represents a strategic advantage for those therapies that involve macrophages. Such is the case of tumor‐associated macrophages (TAMs), which can reprogram/repolarize their predominantly immunosuppressive and tumor‐supportive phenotype toward an immunostimulatory and anti‐tumor phenotype using sEV as nanocarriers of TAMs reprogramming molecules. In this design, sEV represents an advantageous delivery system, providing precision to the therapy by simultaneously matching their tropism to the therapeutic cell target. Here, we review the current knowledge of the role of TAMs in the tumoral microenvironment and the effect generated by the reprogramming of these phagocytic cells fate using sEV. Finally, we discuss how these vesicles can be engineered by different bioengineering techniques to improve their therapeutic cargo loading and preferential uptake by TAMs.

## 1 INTRODUCTION

Tumor‐associated macrophages (TAMs) are an important component of the leukocyte infiltrate of tumors that play a multi‐functional role in cancer progression, exerting dramatic impacts on tumor initiation and promotion, metastasis, immune regulation, and angiogenesis.^1^, ^2^ Derived from circulating inflammatory monocytes and tissue‐resident macrophages that are recruited to tumor tissue and then induced to acquire pro‐tumoral phenotypes, TAMs are characterized by an M2‐like phenotype.^3^ Through the secretion of an array of growth factors, cytokines, chemokines, hormones, matrix‐remodeling proteases, metabolites, and small extracellular vesicles (sEV),^4^ TAMs are not only involved in cancer progression and metastasis but also in the tumor recovery after cytoreductive therapies such as chemotherapy, radiotherapy, and biological therapies.^5^ As Cassetta and Pollard discuss in their review manuscript,^5^ macrophage infiltration also interferes with the immunotherapy efficacy, neutralizing efforts to reactivate CD8+ T cells in some tumors. In clinical studies, an increase in macrophage density in tumors correlates with markers of poor prognosis and reduced overall patient survival, with some exceptions such as colorectal carcinoma.^6^, ^7^

Although several therapeutic strategies have been developed to target macrophages in the tumor microenvironment (TME) at the level of recruitment, survival, or reprogramming toward anti‐tumoral activities,^8^ they are still under preclinical and clinical evaluation. Reprogramming TAMs toward an M1‐like anti‐tumoral phenotype provides the opportunity to balance the leukocyte infiltrate of the tumor stroma, blunting it from a predominantly pro‐tumoral state to one with anti‐tumoral activity, without the drawback of long‐term toxicity caused by the depletion of all macrophages.^5^, ^6^ With promising preclinical results, molecules such as PI3Kγ and histone deacetylases (HDAC) inhibitors have achieved the reprogramming of TAMs toward an anti‐tumor phenotype state, resulting in a decrease in tumor growth together with increased tumor sensitization to immune checkpoint inhibitors,^9^ data that demonstrate the potential clinical efficacy of this therapeutic strategy.^5^, ^6^ However, the main barrier to the clinical translation of TAMs reprogramming strategies is the inadequate drug delivery efficiency, consequence of the aberrant vascular architecture, high interstitial fluid pressure, and the compact structure of the extracellular matrix (ECM) in the tumor.^10^ Nevertheless, these in vivo delivery barriers could be resolved using specialized drug‐delivery methods,^11^, ^12^ such as nanoencapsulation, since it is known that nanoparticles or nanovesicles of ~100 nm in size are selectively retained in tumor tissue through the mechanism called enhanced permeation and retention (EPR) effect.^13^, ^14^

sEV are considered effective nanocarriers of biological origin, both endogenous molecules and those artificially loaded.^15^ Characteristics such as their rapid internalization into acceptor cells, lack of immunogenicity even at repeated doses, and the potential for modifying their molecular cargo or their surface membrane for targeting a specific cell or tissue, have turned sEV into powerful therapeutic agents.^16^ However, the data derived from the pharmacokinetics studies have reported that exogenous or therapeutic exosomes injected intravenously in rodents accumulate primarily in the liver, spleen, lungs, and gastrointestinal tract, uptake attributed mainly to macrophages resident in these organs.^17^ This natural “tropism” of sEV toward monocytes/macrophages represents a barrier to therapeutic strategies that use sEV as nanocarriers of natural or artificially loaded therapeutic molecular cargo by decreasing their bioavailability in the circulation to just a few minutes.^18^, ^19^, ^20^, ^21^ Nonetheless, this drawback can be turned into a strategic advantage to TAMs re‐education in solid tumors, mainly in those with a high infiltrate of myeloid cells. Taking advantage of these native tropisms that results in a rapid and preferential distribution to macrophages, sEV in their native or engineered state can be used as agents to reeducate these phagocytic cells toward a predominantly pro‐inflammatory M1‐like phenotype to regulate tumor progression or favor the response of other therapeutic interventions.^8^ This sEV‐based therapeutic approach has been successfully tested at the preclinical level in a colon carcinoma model. By functionalizing the sEV surface membrane with an antisense oligonucleotide (ASO) targeting the transcription factor STAT6, the reeducation of TAMs from their native M2‐like phenotype to an M1‐like phenotype with potent antitumor properties is achieved.^22^

In this review, we summarize the role of TAMs in the TME and the clinical relevance of these cells as a potential therapeutic target. Specifically, we discuss how the tumor‐supportive phenotype of TAMs can be reprogrammed using engineered sEV as a drug delivery nanocarrier. For this purpose, we present different experimental techniques that modify the cargo and surface of sEV and candidate therapeutic molecules with a reported ability to reprogram TAMs in a wide variety of cancer models.

## 2 PLASTICITY AND DIVERSITY, THE CURRENT MACROPHAGE PERSPECTIVE

Macrophages are myeloid cells from the innate immune system that have an essential role in tissue homeostasis, regeneration, inflammatory response, and elimination of pathogens, among others. Originally, it was thought that macrophages shared their origin with macrophage dendritic cell precursors and bone marrow (BM)‐derived peripheral monocytes; however, during the last years, murine models have demonstrated that different tissue‐resident macrophages populations develop from hematopoietic progenitors present in the yolk sac independently from the BM.^23^, ^24^

Macrophages are highly plastic cells that can integrate multiple signaling pathways simultaneously depending on the stimuli, altering their transcriptional networks and function through epigenetic modifications.^25^ Complementarily, today it is well established that macrophages can reprogram their transcriptional profile by altering only their microenvironment stimuli, results that have been validated both in vitro and in vivo.^26^ The array of functional phenotypes by which these cells respond to the environment changes is known as the macrophage polarization process^.^ ^27^ Since in preclinical and clinical studies the coexistence of different, unique, and even mixed polarization states had been reported a dynamic and reversible mechanism is suggested to regulate the macrophage polarization.^28^ Thanks to the advancement of single‐cell techniques, new evidence supports the presence of a wide diversity of macrophage subpopulations within the same tissue, where different subpopulations regulate physiological and pathological processes.^29^ For these reasons, it is increasingly challenging to attribute a function to a certain phenotype, highlighting the need for the nomenclature in the study of these myeloid cells.

In the early 2000s, the M1/M2 concepts were introduced based on the ability to produce NO in the presence of LPS in vivo.^30^ Nowadays, this binary nomenclature is applied in more generalized ambits, where different populations of macrophages are cataloged with these terms. Even though this overgeneralized classification misses the macrophage diversity, it is still one of the most common nomenclatures in the literature. Another nomenclature in macrophages is based on the activation stimuli and the expression of certain markers.^31^ In this sense, the classical activation pathway corresponds to the incubation of macrophages with IFN‐γ and LPS in vitro, inducing the differentiation to M1‐like macrophages with increased production of NO through the expression of NOS2, increased antibody‐dependent cellular phagocytosis (ADCP), membrane concentration of MHCII and secretion of pro‐inflammatory cytokines (IL‐1, TNF, IFN), among others.^31^ Instead, the alternative activation pathway that originates the M2‐like macrophages corresponds to the incubation with IL‐4 in vitro, increasing the expression of Arg1 (inhibitor by competition of NOS), the secretion of anti‐inflammatory cytokines (IL‐4, IL‐10, IL‐13, among others), and immunosuppressive and angiogenic factors.^31^

## 3 ROLE OF TUMOR‐ASSOCIATED MACROPHAGES IN HUMAN CANCER

The TME is a complex ecosystem in which tumor cells coexist with a heterogeneous cell population composed of multiple cell types like endothelial cells, stromal cells, and immune cells.^32^ Macrophages, polymorphonuclear cells, mast cells, natural killer, dendritic cells, and T and B lymphocytes are the immune cell components that essentially determine the tumor's fate and the progression of the metastatic disease.^33^ In fact, the immune cell composition and organization within the TME are correlated to clinical outcomes in cancer patients.^33^ TAMs and their precursors are the most abundant population of the tumor‐infiltrating immune cell in many solid tumors, as shown by immunohistochemical analyses of the TAMs marker CD68^+^ and by CIBERSORT‐mediated dissection of gene expression profile.^5^, ^34^, ^35^ TAMs are recruited into the tumor by chemokines secreted by cancer and stromal cells, playing an essential role in the regulation of cancer‐related inflammation.^36^ Different subpopulations of TAMs act as a source of local and systemic cues to support the tumor angiogenesis, proliferation, survival, and invasiveness of tumor cells; and suppression of cytolytic T‐cell responses.^35^ This assortment of pro‐tumor functions is consistent with the results of clinical studies showing that human macrophage density in tumor samples is associated with poor prognosis.^37^ In the last 5 years, the correlation between TAM infiltration and progression in cancer has been confirmed by several meta‐analyses performed in breast cancer,^38^ cervical carcinoma,^39^ gastric cancer,^40^ Hodgkin lymphoma,^41^ pancreatic cancer,^42^ and lung cancer^43^ but not in colorectal cancer.^44^

Like macrophages in normal tissues, TAMs are characterized by their great phenotypic and functional plasticity that allows them to adopt a pro‐inflammatory M1‐like state, or an anti‐inflammatory M2‐like state or even an intermediate activation state depending on the environmental conditions to which they are exposed locally in the TME or those that occur during a treatment.^5^, ^34^, ^45^ Furthermore, a recent analysis of the tumor‐immune signature shows that different subpopulations of TAMs that coexist in the TME often co‐express canonical pro‐inflammatory M1 and alternatively activated M2 genes in individual cells.^34^, ^46^, ^47^, ^48^ Although the M2‐like pro‐tumoral TAMs is the phenotype predominantly found in tumors, in the very early phases of oncogenesis the foremost polarization of the TAMs is toward the M1‐like state, which mediates multipronged anticancer effects.^34^, ^36^, ^49^ However, this permanent state of pro‐inflammatory activity induces genomic instability in cancer cells, which in consequence acquire the ability to repolarize TAMs toward an M2‐like state.^50^ This repolarization of TAMs favors tumor progression and malignancy through the secretion of growth factors, cytokines, and chemokines—such as transforming growth factor β (TGF‐β), vascular endothelial growth factor (VEGF), platelet‐derived growth factor (PDGF), M‐CSF, IL‐10, and chemokine C‐X‐C motif ligand (CXCL)^51^—hormones, matrix‐remodeling proteases, metabolites, and sEV.^4^, ^37^

**FIGURE 1:**
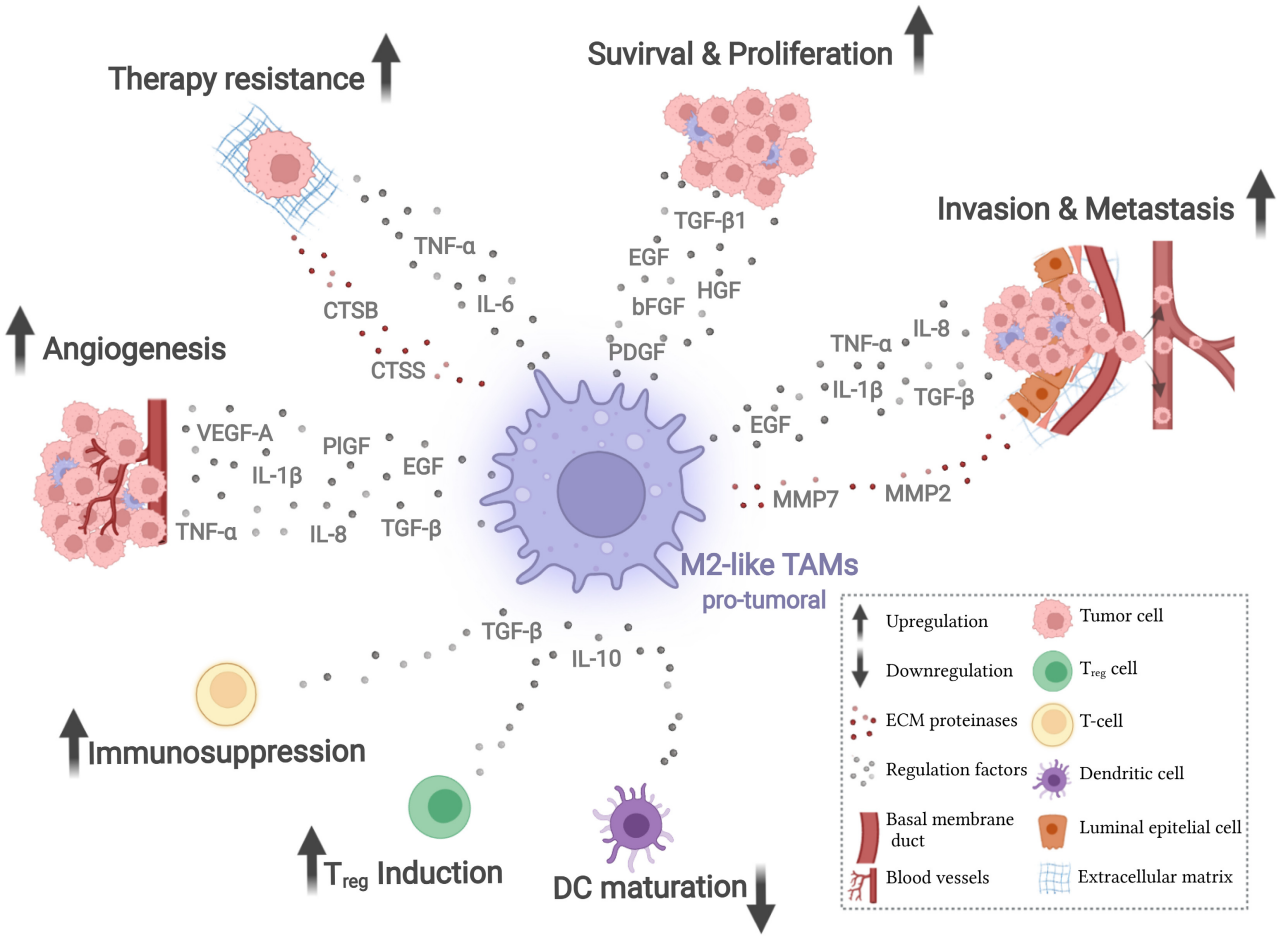
The pro‐tumor phenotype of TAMs promotes tumor development through multiple interactions with TME. TAMs correspond to the most abundant infiltrating immune cell population in TME, exerting various actions in favor of carcinogenesis. Through direct and indirect interactions (through the secretion of growth factors, cytokines, and interleukins, among others) with the other infiltrating cell populations in the TME, the pro‐tumor phenotype of TAMs contributes to greater survival and proliferation of tumor cells, as well as in the promotion of malignant characteristics such as invasion and metastasis. By located in predominantly hypoxic regions in the TME, TAMs also have strong pro‐angiogenic properties that help support tumor development and progression. Also, TAMs create an inflammatory and immune‐evading environment, interfering with the response to antitumor treatments, especially immunotherapy. CTSB, cathepsin B; CTSS, cathepsin S; ECM, extracellular matrix; EGF, epidermal growth factor; HGF, hepatocyte growth factor; IL‐10, interleukin 10; IL‐1β, interleukin 1 beta; IL‐6, interleukin 6; IL‐8, interleukin 8; MMP2, matrix metalloproteinase 2; MMP7, matrix metalloproteinase 7; PDGF, platelet‐derived growth factor; PIGF, placenta growth factor; TGF‐β1, transforming growth factor‐beta 1; TNF‐α, tumor necrosis factor‐alpha; VEGF‐A, vascular endothelial growth factor A (created with BioRender.com)

A distinct subpopulation of TAMs with different transcriptome and cell surface markers stimulates different aspects of carcinogenesis such as angiogenesis, immune evasion, invasion, and metastasis^2^, ^6^, ^37^, ^52^, ^53^ (Figure 1). However, its presence in TME also regulates the response to treatments. In vivo studies showed that the pro‐tumoral phenotype of TAMs mediates acquired resistance of cancer cells to chemotherapy and radiotherapy providing them with survival factors that activate anti‐apoptotic programs in malignant cells.^51^ In fact, selective depletion or inhibition of TAMs results in a reduction of the resistance associated with chemo and radiotherapy.^51^ Furthermore, TAMs have also been considered regulators of immunotherapy since the immunosuppression they cause acts as a barrier to tumor sensitivity to cancer immunotherapies, thus inducing acquired resistance and immune evasion of the tumor. Although the mechanisms contributing to the immunotherapy resistance are still under investigation, it is known that macrophages express elevated levels of CTLA‐4 (an immune checkpoint receptor), which are associated with the downregulation of anti‐tumor activities of T‐cells.^54^ In addition, CD68+ macrophages are the predominant immune cells expressing PD‐L1,^55^ an immune regulatory molecule that interacts with PD‐1 on T cells at the immunological synapse. Activated by TME‐derived factors, these PD‐L1+ TAMs mediate CD8+ T‐cell dysfunction through the PD‐1/PD‐L1 axis, observations reported in several types of cancer as hepatocellular carcinoma,^56^ ovarian cancer,^57^ bladder cancer,^58^ soft tissue sarcoma,^59^ head, and neck squamous cell carcinoma,^60^ and cholangiocarcinoma.^61^ Also, TAMs express various other checkpoints ligands such as PD‐1,^62^ PD‐L2,^63^ B7‐S1,^64^ galectin‐9,^65^ and V‐domain Ig‐containing suppressor of T‐cell activation (VISTA)^66^ that inhibit the anti‐tumor immune response at different levels.^67^

## 4 TUMOR‐ASSOCIATED MACROPHAGES AS THERAPEUTIC TARGETS

Since TAMs play a crucial role in the regulation of cancer‐related inflammation and interfere with the beneficial outcomes of current therapeutic strategies, TAMs represent a target cell population in new anti‐tumor therapeutic approaches. Other features like its great abundance in the tumor, its genomic stability, and rapid response to external stimuli reflected in its extreme plasticity highlight its potential as a therapeutic target for cancer.^8^ Reprogramming TAMs toward an M1‐like anti‐tumor phenotype leads to a rebalancing of the composition of TAMs in TME toward a predominantly pro‐inflammatory population that limits tumor progression and increases anti‐tumor immune responses.

One of the first studies to show that TAM re‐education within TME exerts an effect on tumor regression was reported in 2013, using a colony‐stimulating factor 1 receptor (CSF‐1R) inhibitor—called BLZ945—in multiple preclinical glioblastoma models.^68^ After the inhibition of CSF‐1R, the authors observed a blockage in the growth and progression of the glioma, however, surprisingly this anti‐tumor effect was not mediated by the depletion of TAMs but rather by their re‐education within the glioma microenvironment. Unlike macrophage depletion, which was observed in healthy tissues (including the brain), TAM survival was mediated by factors secreted by the tumor itself, such as granulocyte‐macrophages CSF (GM‐CSF) and interferon‐γ (IFN‐γ). These glioma‐supplied cytokines were also responsible for changing the transcriptomic profile of surviving TAMs, reprogramming them toward a less pro‐tumoral phenotype by reducing the polarization of M2 macrophages in gliomas treated with CSF‐1R inhibitor. In line with this study, research led by Joyce et al.^69^ demonstrated that the CSF‐1R inhibitor PLX3397 (chemically distinct from BLZ945) also induces the repolarization of TAMs from an M2‐like state, the mechanism that restores the sensitivity of glioma cells to tyrosine kinase inhibitors in preclinical glioblastoma model.

The dual agonists of the toll‐like receptors TLR7/8—such as resiquimod (R848) or imiquimod—are drugs widely recognized for their ability to promote the M2–M1 transition. These activating agents of the immune system induce a profound shift toward an anti‐tumor immune state in the immune cells that cohabit in TME, stimulating the representation of M1‐like macrophages in colorectal cancer,^70^ triple‐negative breast cancer,^71^ and melanoma.^70^ Interestingly, it has also been reported that these agonists of TLR7/8 enhance the anti‐tumor efficacy of some antibodies or chemotherapeutic agents, currently being studied in combined therapeutic strategies. However, their systemic administration causes severe cytokine release syndrome and systemic autoimmunity, which has limited their clinical translation.^72^ For this reason, in recent years, several researchers have used nanocarriers for the safe and efficient transport of these agonists TLR7/8. For example, Rodell et al. demonstrated that R848‐loaded β‐cyclodextrin nanoparticles (CDNP‐R848) lead to an efficient administration of the drug to TAMs, inducing an M2–M1 transition that allows controlling tumor growth in vivo.^70^ In the same line, Figueiredo et al. demonstrated that lignin nanoparticles modified the biodistribution of R848, allowing an increase in its tumor accumulation and effectiveness in reverting the pro‐tumor phenotype of macrophages into M1‐like anti‐tumor macrophages in TME which was not achieved by systemic administration of free R848.^71^

Recently, Wang et al.^73^ also described an interesting TAM reprogramming strategy evaluated at the preclinical level but with an interesting translational approach. Using a nucleic acid carrier system with an affinity for mannose receptors in TAMs/tumor‐infiltrating dendritic cells (TIDC) and that specifically responds to the low‐pH tumor microenvironment,^74^ the authors achieve TAM‐targeted delivery of miR‐99b and/or miR‐125a. After the effective transfer of these miRNAs to TAMs, the growth of an orthotopic tumor of murine hepatocellular carcinoma (HCC) and subcutaneous Lewis's lung cancer (LLC) was significantly prevented by re‐education of TAMs toward an anti‐tumor phenotype (M1‐like state) with enhanced immune surveillance.^73^ The studies of the mechanism of action revealed that miR‐99b might promote M1 while inhibiting M2 polarization by downregulating the signaling pathways of κB‐Ras2 and/or mTOR, respectively. Interestingly, the authors reported that miR‐99b might amplify M1 macrophage function through NF‐κB by a positive feedback regulation loop, resulting in increased phagocytosis and antigen presentation.

Other immunomodulatory agents such as interleukin‐12 (IL‐12)^75^ or CD40 agonists^76^ have also been reported to induce repolarization of TAMs. However, since these immunomodulatory molecules can activate different cell types, they are associated with dose‐limiting adverse effects and systemic toxicities.^77^ Other strategies used to repolarize macrophages are described in Table 1, which summarizes the main biological findings in a cancer context. Given that in the literature it is possible to find several review articles that describe in detail the different molecules that exert their effect in the re‐education of TAMs, we present in Table 1 those strategies described from the year 2018 onwards.

**TABLE 1:** Molecules that induce a TAMs re‐education

References	Molecule/construct	Target	Biological effect	Cancer model	Type of study
*TLR agonists*					
147	Resiquimod	TLR7/8	Increased antibody‐dependent cellular phagocytosis	Colorectal adenocarcinoma	In vitro and in vivo
148	Poly(I:C) and Imiquimod	TLR3 and TLR7	Increased expression of MHCII	Pancreas	In vitro
149	CpG oligodeoxynucleotides	TLR9	Increased secretion of TNF and IL‐6 and decreased IL‐10	Breast	In vitro and in vivo
*RNAs*					
73	miR‐99b	mTOR and κB‐Ras2	Induction of phagocytosis, antigen presentation, and secretion of pro‐inflammatory cytokines	Hepatocellular and Lewis lung carcinoma	In vitro and in vivo
150	miR‐125b	IRF4	Decreased ascitic fluid accumulation and VEGF secretion	Ovarian and nonsmall‐cell lung cancer	In vitro and in vivo
151	IKKβ‐siRNA	IKKβ	Decreased secretion of TGF‐β and IL‐10 and increased of IFN‐γ and IL‐12	Hepatocellular carcinoma	In vitro and in vivo
*Saccharides*					
152	Mannose	CD206	Increased CD86/CD206 ratio	Breast and Glioblastoma	In vitro and in vivo
153	*Lepidium meyenii Walp*. polysaccharide and doxorubicin	Suggested to be TLR4	Activation of p65 and STAT1 and STAT3 inhibition	Breast	In vivo
154	*Astragalus membranaceus* polysaccharide *(PG2)*	Suggested to be NF‐κB pathway	Decreased anti‐inflammatory cytokines and increased CD86/CD206 ratio	Lung	In vitro and in vivo
155	Chitooligosaccharides	Non‐described	Increased CD86/CD206 ratio and IL‐12 secretion	Liver	In vitro and in vivo
156	*Ilex asprella* acidic polysaccharide	NF‐κB and STAT1/3 signaling pathways	Decreased VEGF and IL‐10 secretion with higher MHCII expression	Sarcoma	In vitro and in vivo
*Antibodies*					
157	Anti‐tumor IgE (SF‐25) and anti‐human IgE crosslink	FcεRI	Increased pro‐inflammatory cytokine secretion	Melanoma	In vitro and ex vivo
158	Murlentamab	AMHRII	Increased antibody‐dependent cytotoxicity	Ovarian	In vitro
*Small molecules*					
159	3‐Methyladenine (3‐MA)	P13K	Increased CD86/CD206 ratio and secretion of pro‐inflammatory cytokines	Breast	In vitro and in vivo
160	Astragaloside IV	AMPK signaling pathway	Increased expression of pro‐inflammatory factors and MHCII	Colorectal	In vitro and in vivo
161	AZD5069	CXCR2	Increased secretion of TNF‐α and a decrease in tumor vasculature	Prostate	In vivo
162	AZD5153	BRD4	Blockage of IL‐10 secretion and increased IL‐12	Ovarian	In vitro and in vivo
163	CARP‐1 functional mimetic (C4.16) and Sorafenib	AKT‐kinases	Increased CD86/CD206 and iNOS/Arg1 ratios	Renal carcinoma	In vitro
164	IMD‐0354	IKKβ	Increased CD86/CD206 ratio	Hepatocellular and colon carcinoma	In vitro and in vivo
165	IPI549	PI3Kγ	Increased iNOS expression and decreased CD206	Breast	In vivo
166	Rapamycin and hydroxychloroquine	mTOR	Increased expression of IL‐1 and TNF‐α together with a decrease of CD206 and CD163 expression	Glioblastoma	In vitro and in vivo
167	Regorafenib and disulfiram/copper complex	Multikinases	Increased TNF‐α secretion, decrease in TGF‐β	Glioblastoma	In vitro and in vivo
168	Resveratrol	NF‐B pathway	Increased CD86/CD206 ratio, iNOS expression, and decreased IL‐10 secretion	Breast	In vitro and in vivo
169	Simvastatin	LXR/ABCA1	Decreased expression of CD206 and TGF‐β and increased expression of pro‐inflammatory cytokines	Nonsmall‐cell lung cancer	In vitro and in vivo
170	Simvastatin and gefitinib	EGFR/Akt/Erk signaling pathway	Decreased expression of CD206 and TGF‐β	Glioma	In vitro and in vivo
171	Vismodegib	Hedgehog pathway	Decreased expression of Arg1 and CD206	Breast	In vitro and in vivo
172	Vorinostat and simvastatin	STAT6 and LXR/ABCA1	Increased CD80/CD206 ratio and TNF‐α secretion	Nonsmall cell lung cancer	In vitro and in vivo

## 5 RE‐EDUCATION OF TUMOR‐ASSOCIATED MACROPHAGES USING SEV

Low drug solubility, nonspecific biodistribution, short half‐life, and poor cellular uptake are some of the main in vivo delivery barriers that hamper the efficacy of agents targeting TAMs.^12^ Nanoencapsulation of drugs has proven to be a methodology that allows increasing the accumulation of these molecules in tumors through an EPR effect that results in better therapeutic efficacy with reduced systemic toxicity.^13^, ^14^ In addition, this method decreases the interaction of the drug with transport proteins and binding agents in serum, important factors that modulate the activity and biodistribution after intravenous administration.^78^ There are specialized drug‐delivery methods, such as liposomes, polymeric nanoparticles, inorganic nanoparticles, and biological or natural carriers as sEV that have demonstrated their efficacy and safety in the transport and delivery of the therapeutic molecular material that they encapsulate to target cells or specific tissue.^12^ Among them, sEV are the nanovesicles that are currently revolutionizing the field of biomedicine due to their numerous advantages as carriers of therapeutic molecules. Attributes as their stability, biocompatibility, permeability, low toxicity, and low immunogenicity determine its success as a nanoparticle drug delivery system.^16^

sEV are non‐self‐replicative lipid‐based vesicles generated from different subcellular compartments with a demonstrated role in cell–cell communication.^15^ Characterized by a size smaller than 200 nm and with membrane expression of the tetraspanins CD63, CD81, and CD9, sEV internalize into acceptor cells with extraordinary ease due to the lipid and protein composition of their membrane, which is very similar to the cell membrane. Detailed proteomic and biochemical analyses have shown that sEV contain, transport, and protect a multitude of molecules such as lipids, proteins, nucleic acids, and metabolites among others, cargo once transferred to the different acceptor cells, triggers effective phenotypic changes in them, as first described by Valadi et al.^79^

Several preclinical investigations have demonstrated the feasibility of using sEV as carriers of natural or artificially loaded therapeutic molecules. In diseases such as cancer,^80^, ^81^, ^82^, ^83^ chronic kidney disease,^84^ osteoarthritis,^85^ idiopathic pulmonary fibrosis,^86^ and myocardial infarction,^87^ its ability to slow, stop or even reverse the progression of the disease has been observed, along with demonstrating good tolerability to its local or systemic administration, including repeated dosing.^88^ Nonetheless, biodistribution and pharmacokinetic studies in murine models have reported that after systemic administration, therapeutic exosomes rapidly disappear from the blood circulation in a half‐time of ~2–30 min.^16^, ^18^, ^19^, ^20^, ^21^ In fact, these studies showed that after 2 min of intravenous injection ~30% of sEV remained in circulation, bioavailability decreases to 1.8%–3.3% between 5 and 30 min later to a minimum of 1.4%–0% after 1 h of systemically administered sEV.^89^ In a nonhuman primate model (*Macaca nemestrina*), sEV labeled with a highly sensitive nanoluciferase reporter (palmGRET) administered intravenously have markedly longer circulation times in plasma than previously reported in mice, reaching a half‐time in plasma of ~36–42 min.^90^ Macrophages associated with the organs of the mononuclear phagocytic system (liver, spleen, and lung) are mainly responsible for this clearance since the negative charge of phosphatidylserine ‐which is enriched in the surface of the sEV—activates the “eat‐me” signal in these phagocytic cells limiting their bioavailability and therapeutic potential.^91^, ^92^, ^93^ Other molecules such as the scavenger receptor class A family (SR‐A),^94^ lectins,^95^, ^96^ sialic acid,^97^ and the activation of the complement systems have also been involved in the rapid clearance of sEV in murine models; and, in nonhuman primates, CD20+ B cells and CD3+ cells have also been identified as important contributors in the uptake of sEV.^90^ Despite this, various research works have demonstrated that unmodified sEV administered systemically manages to reach the tumor tissue in a variable percentage depending on the source of the parent cell, the type and anatomical location of the tumor, and the technique used to label the sEV.^17^, ^94^, ^98^ For example, it has been described that after 24 h of systemically injected HEK293T DiR‐labeled sEV, ~3% of the total tissue fluorescence was detected in the tumor in a melanoma murine model.^17^ Likewise, unpublished data from our laboratory also shows that DiR‐labeled sEV derived from mesenchymal stem cells possess the ability to localize the breast tumor tissue and accumulate in it with a 30‐fold increase compared to the healthy contralateral mammary tissue in vivo.

Although the sEV–macrophage interaction clearly represents an obstacle to the bioavailability of a therapy based on sEV, this natural tropism can be used strategically for the selective delivery of reprogramming molecules of TAMs, especially in tumors rich in myeloid cells such as glioma, lung adenocarcinoma, hepatocellular carcinoma, pancreatic ductal adenocarcinoma, colorectal cancer, ovarian cancer, among others. Selective delivery of a natural or artificially loaded cargo to TAMs would allow their re‐education toward a predominant M1‐like state, promoting a remodeling of the TME and the potential establishment of an adaptive anti‐tumor response mediated by T‐cells. Several reports have demonstrated that sEV of different cellular origins possess the potential to switch the fate of macrophage phenotypes in TME, differentiating them into alternatively activated pro‐tumorigenic M2‐like macrophage^99^, ^100^, ^101^, ^102^ or classically activated anti‐tumorigenic M1‐like macrophage.^103^, ^104^, ^105^, ^106^, ^107^, ^108^, ^109^

Specifically, regarding polarization toward an anti‐tumor M1‐like phenotype, it has been reported that sEV derived from murine H22 hepatoma cell line used as vehicles to carrier iron oxide nanoparticles achieve M1‐like macrophages polarization, which inhibits the hepatocellular carcinoma growth in mice.^117^ In the same line, the research carried out by Moradi‐Chaleshtori et al.^104^, ^105^ demonstrated that the treatment with sEV enriched in miR‐130 and miR‐33 induces in macrophages the upregulation of M1‐specific markers such as CD86, IRF5, NOS2, TNF‐α, and IL‐1β, and downregulation of M2 specific markers as CD206, Ym1, Arg1, TGF‐β, and IL‐10. Also, the authors observed a greater ability of phagocytosis of macrophages, while in breast tumor cells it was observed a reduction in their migration and invasion properties after reprogramming of macrophages.^104^, ^105^ In an in vivo setting, the authors also observed that macrophage polarization toward their M1‐like state consistently induced an inhibitory effect on breast tumor growth in mice.^105^ In colorectal cancer (CRC), it has been observed that miR‐21‐containing sEV derived from CRC cells possess a tropism toward liver tissue, where they induce in macrophages resident (Küpffer cells) a polarization toward an IL‐6‐secreting proinflammatory phenotype^106^ (Table 2).

**TABLE 2:** sEV used as TAM repolarization agents

References	Parental cell of sEV	sEV modification	sEV cargo	Macrophage Origin	Phenotype induced	Cancer type	Biological effect	Type of study
98	H22	Modified	PIONs@E6	mPBMCs	M1‐like	Hepatocarcinoma	Detrimental primary tumor growth	In vitro In vivo
99	4 T1	Modified	miR‐130 mimic	Mouse peritoneal macrophages	M1‐like	Breast	Suppress breast tumor cell invasion and migration	In vitro
100	MDA‐MB‐231 MCF‐10A	Modified	miR‐130 miR‐33	hPBMCs	M1‐like	Breast	Suppress tumor growth and cell invasiveness	In vitro In vivo
101	SW480 SW620 LoVo	Unmodified	miR‐21	THP‐1 RAW264.7	M1‐like	Colon	Pro‐metastasic	In vitro In vivo
102, 103	–	Modified	ASO‐STAT6	–	M1‐like	Colon & hepatocarcinoma	Tumor growth inhibition	In vivo
104	BM‐MSC	Modified	Galactin‐9 siRNA and surface modified oxaliplatin	Mouse peritoneal macrophages	M1‐like	Pancreatic ductal adenocarcinoma	Tumor growth inhibition	In vivo

Abbreviations: 4T1, murine breast cancer cell line; ASO‐STAT6, antisense oligonucleotide targeting STAT6; BM‐MSC, bone marrow mesenchymal stem cells; CL1‐5, lung cancer cells; H22, murine hepatoma cell line; HCT116, human colorectal carcinoma cell line; hPBMCs, human peripheral blood mononuclear cells; MDA‐MB‐231 and MCF‐10A, human breast cancer cell line; mPBMCs, murine peripheral blood mononuclear cells; PANC‐1, pancreatic cancer cell line; PIONs@E6, pegylated IONs loaded with chlorin e6; RAW264‐7, murine macrophage cell line; SW480, SW620, and LoVo, human colon cancer cell line; THP‐1, human monocytic leukemia cells.

In a series of studies, Kamerkar et al.^22^, ^107^, ^108^ have shown that sEV loaded with ASO (ExoASO) targeting STAT6 or C/EBPβ, key transcription factors that control the immunosuppressive program of TAMs, induces macrophage reprogramming to a pro‐inflammatory M1‐like phenotype which results in a potent‐single agent anti‐tumor activity in multiple checkpoints refractory tumor models. Specifically, these studies show that ExoASO STAT6 or C/EBPβ induces silencing of STAT6 and C/EBPβ mRNA transcripts, respectively, in primary human M2‐like macrophages with greater potency than the free ASO. Through gene expression analysis and cytokine assays, the authors observed reprogramming of M2‐like to M1‐like macrophages, determined by a decrease in CD163 and TGF‐β1 expression, and consequent induction of the pro‐inflammatory cytokines TNFα and IL‐12p40. In in vivo efficacy studies in colon carcinoma CT26 tumors, ExoASO STAT6 or C/EBPβ treatment results in a reduction in the expression of STAT6 and C/EBPβ in tumor‐associated CD11b+ myeloid cells. Likewise, consistent with effective macrophage reprogramming, the authors reported modulation of CD206 (also known as macrophage mannose receptor 1, MRC1), CSF1r, and iNOS. As monotherapy, ExoASO‐STAT6 and ExoASO‐C/EBPβ showed significant anti‐tumor activity.^107^ In an orthotopic model of HCC resistant to anti‐PD‐1 or anti‐CSF1R immunotherapies, systemic administration of ExoASO‐STAT6 also results in a potent anti‐tumor activity associated with a decrease in M2‐like markers as TGF‐β1 and Ccl17, an increase in the M1‐like marker IL‐1β, and an increase in interferon (IFN) and cytotoxic T‐cell gene signatures consistent with a reprogramming of the TME.^108^

Recently, it has been reported an sEV‐based dual delivery biosystem enhances the response to immunotherapy and reprogramming the TME toward one with a less suppressive environment in pancreatic ductal adenocarcinoma (PDAC) preclinical model.^109^ Using sEV derived from BM‐mesenchymal stem cells to improve tumor targeting efficacy, the authors construct iEXO‐OXA through the loading of galectin‐9 siRNA to reverse immunosuppression caused by M2‐like cells and superficially modified with oxaliplatin (OXA) prodrug as immunogenic cell death (ICD)‐trigger. After treatment with iEXO and iEXO‐OXA, tumors showed a reduction in the M2‐like TAMs population (CD45+ CD11b+ CD206+) and an increase in M1‐like TAMs (CD45+ CD11b+ CD16/32+), suggesting an improvement of the immunosuppressive TME allowing a greater infiltration of antitumoral cytotoxic T lymphocytes.

## 6 ENGINEERED sEV TO SELECTIVELY REEDUCATE TAMS TOWARD ANTI‐TUMOR ACTIVITY

Enriching sEV with specific reprogramming molecules to efficiently promote reeducation of TAMs toward an M1‐like phenotype is a plausible strategy to achieve TEM reform toward one with pro‐inflammatory activity permissive to the action of the immune system. Indeed, this strategy should be designed to impact TAMs preferentially or selectively, without affecting circulating monocytes or tissue‐resident macrophages, to limit the potential side effects due to an off‐target interaction. Both loading with specific molecules to achieve the desired therapeutic efficacy and selective targeting to TAM to ensure therapeutic safety are the fundamental pillars on which modifications of sEV should be designed. Fortunately, native sEV can be modified both in their molecular cargo and in their surface, membrane using different biotechnological tools with variable influence on the integrity of the membrane or the bioactivity of the therapeutic payload.

### 6.1 Enrichment of sEV with TAM reprogramming molecules

As shown in Table 1, there are countless studies that report molecules with the potential to trigger reeducation of TAMs toward a pro‐inflammatory phenotype. Indeed, the efficacy of these drugs can be potentially improved through a delivery platform using sEV that allows their accumulation in tumor tissue due to the EPR effect. These therapeutic molecules can be enriched into sEV, either by “loading” them inside or onto their surface to achieve controlled and efficient delivery to the tumor niche. Several excellent manuscripts can be found in the literature that review these different methodological techniques in‐depth,^110^, ^111^, ^112^, ^113^ so here we will only analyze them briefly, presenting the advantages and disadvantages of each of them (Table 3). In general terms, “loading” techniques seek to enrich the cargo of sEV or on their surface with specific endogenous or exogenous molecules of natural or artificial origin (as small molecules, drugs, proteins, or nucleic acids) to increase their effective transfer to the acceptor cell or target tissue to trigger a desired biological effect. These techniques can be classified into two major approaches: pre‐ and post‐isolation techniques. In the pre‐isolation techniques or cell‐based loading methods, the starting material is the parental cells; therefore, the enrichment with a specific cargo occurs before the isolation of the sEV. On the contrary, in the post‐isolation techniques or noncell‐based loading methods, sEV are the direct starting material for specific molecular enrichment; thus, modifications occur after isolation of sEV by ultracentrifugation, gradient ultracentrifugation, size‐exclusion chromatography, co‐precipitation, or field flow fractionation.

**TABLE 3:** Advantages and disadvantages of the sEV loading methods

References	Loading method	Approach	Type of cargo	Cargo location in sEV	Advantages	Disadvantages
116	WW tag	Parental cell based	Protein	Lumen	Allows functional/active protein‐specific cargos.	Requires a plasmid construct and transfection.
117	EXPLORs	Parental cell based	Protein	Lumen	Reversible protein–protein interactions.	Requires two transiently transfected vectors and blue light illumination. Involves a plant protein.
120	TAT/TAR	Parental cell based	miRNA	Lumen	Enhanced loading of miRNAs.	Requires a plasmid construct, transfection, and miRNA hairpin structure modification.
124	Three‐way junction (3WJ)	Post isolation	siRNA/miRNA/Protein	Surface/Lumen	Allows directional control of sEV.	Requires previous isolation of 3WJ particles and conjugation of cholesterol for surface location.
173	Lamp2, CD63 or CD9‐based display	Parental cell based	Protein	Surface	Specific targeting of sEV.	Requires molecular cloning and transfection.
174	VSVG‐based display	Parental cell based	Protein	Surface	Improved surface display and sEV uptake in target cells.	Requires cell transfection with VSVG gene encoding construct (is a viral protein).
119	EXOtic	Parental cell based	mRNA	Lumen	mRNA delivery into the cytosol of target cells.	Requires cell transfection with several constructs.
175	Transfection	Parental cell based	DNA/RNA/Protein	Surface/Lumen	May allow a continuous source of modified sEV from a particular cell source.	Requires genetic stable/transiently modification of parental cell. Unspecific sEV loading.
176	Transfection	Post isolation	DNA /RNA/Protein	Lumen	Commercially available kits, straightforward use.	Low loading efficiency; requires separation and purification of sEV after transfection.
141	Click‐chemistry Cloaking Bioconjugation Coating	Post isolation	Targeting peptides/proteins/drugs/biotinylated‐ligand proteins.	Surface	High specificity of loading, pH‐controlled release of drugs, degradable encapsulation of sEV.	Reagent's availability and compatibility with in vivo/in vitro platforms.
177	Electroporation Freeze–thaw Extrusion Sonication	Post isolation	Drugs/nucleic acids/nanomaterials /lipids/proteins	Lumen	Low time requirements.	No provide a continuous source of engineered vesicles. Low loading efficiency, sEV aggregation, and induced sEV membrane damage.
121	Incubation	Parental cell based/Post isolation	Hydrophobic/hydrophilic molecules	Surface/Lumen	Simple and convenient.	Low loading efficiency and cargo quantitation difficult to control. pH can also influence the loading.

#### 6.1.1 Pre‐isolation techniques

Using cell‐based loading approaches, sEV can be modified to load therapeutic proteins directly into their cargo or display them on its surface.^16^ To carry out these modifications, methods that use molecule sorting modules (MSMs) for sorting specific proteins and RNAs to sEV, or a nonspecific sEV enrichment methodology without involving modules for packaging and sorting can be used. Regarding the latter, the genetic editing of sEV‐producing cells is usually used to enrich specific proteins or nucleic acids, a strategy that involves the transfection of the parental cell with a gene of interest.^112^ Likewise, to load specific drugs, direct co‐incubation of the parental cell with the drug allows its encapsulation in the sEV and subsequent secretion.^114^

Regarding the methods that use MSMs, to guide a protein to the surface of sEV, a signal peptide is commonly used. For example, lysosome‐associated membrane protein 2b (Lamp2b) is a sEV surface protein with a signal peptide that has been widely used to display any fusion protein on the surface of sEV as a targeting moiety, ligand, or receptor. Other molecules that have been successfully used for the presentation of fusion proteins on the surface of sEV are the tetraspanins CD63, CD9, CD81; glycosylphosphatidylinositol (GPI); platelet‐derived growth factor receptors (PDGFR); lactadherin (C1C2 domain); and the NH2‐terminus of CD63. For loading therapeutic proteins, existing methods mimic the natural protein sorting system of cells based on ubiquitination. Acting as a sorting signal sequence, ubiquitin tag method has been used to fuse proteins of interest which allows an efficient loading of proteins inside sEV.^115^ WW tag method is based on the labeling of a target protein with ‘WW tag’ that leads to the recognition of the L‐domain‐containing protein Ndfip1, resulting in ubiquitination and loading into sEV.^116^ Other specific methods described for loading proteins into the lumen of sEV include the EXPLORs method (exosomes for protein loading via optically reversible protein–protein interaction‐PPIs)^117^ and the use of nonfunctional mutant of the HIV‐I Nef protein.^118^

For loading therapeutic mRNAs into sEV, a synthetic biology‐inspired control device called EXOsomal Transfer Into Cells (EXOtic) devices functions in an efficient and customizable way to produce engineered sEV. These genetically encoded devices in sEV producer cells enhance the secretion of sEV, specific mRNA packaging, and their delivery into the cytosol of target acceptor cells.^119^ To enrich miRNAs in sEV, Sutaria et al.^120^ developed the system TAT‐TAR protein–RNA interaction strategy that allows to load sEV with any specific (and therapeutic) miRNAs. In this research, the authors fused a pre‐miRNA, to a trans‐activating response element (TAR) sequence (trans‐activation response RNA loop); and, separately, they fused a trans‐activator of transcription (TAT) peptide (transcription activator peptide of HIV‐1) to Lamp2a (Lamp2a‐TAT).^112^ When expressed in cells, miRNA‐TAR binds to Lamp2a‐TAT, and the interaction on the luminal C‐terminal of Lamp2a leads to the effective loading of the miRNA into the lumen of sEV.^112^, ^120^

#### 6.1.2 Post‐isolation techniques

Noncell‐based loading methods use the sEV themselves (after isolation of the parental cells) as starting material to carry out the modifications, either displayed on their surface membrane or their content. Although less complex than pre‐isolation approaches, these techniques certainly require experience working at the nanoscale. As direct loading methods, incubation, sonication, extrusion, electroporation, freeze–thaw treatment, and dialysis are strategies with variable efficiency to load exogenous molecules into sEV (efficiency that depends on the intrinsic properties of the cargo such as hydrophilicity, hydrophobicity, and molecular weight) and with variable alteration of the integrity and functionality of the sEV, attributes that must be verified after making modifications.

Incubation is the most straightforward approach to loading a specific cargo on sEV by diffusion through a concentration gradient. Using particular times, temperatures, and pH, different laboratories have successfully loaded hydrophilic and hydrophobic molecules.^113^ Since sEV contains a hydrophilic core and lipid bilayer in its membrane, hydrophilic drugs tend to incorporate into the aqueous phase within sEV. In contrast, hydrophobic drugs are more stable in the lipid‐enriched membrane. Some molecules that have been successfully loaded into sEV are doxorubicin,^121^ paclitaxel,^122^ curcumin,^123^ withaferin A,^123^ anthocyanidins,^123^ nucleic acids,^121^, ^124^ proteins, and peptides.^125^

**FIGURE 2:**
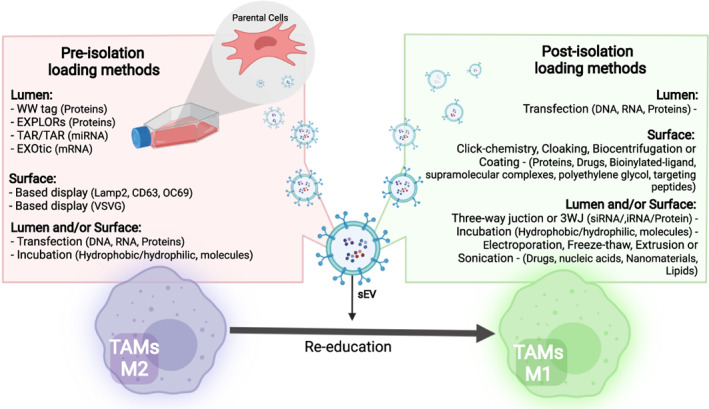
Engineering sEV for targeted drug delivery. Small extracellular vesicles are a versatile platform for drug encapsulation, possessing multiple advantages over conventional synthetic nanocarriers for drug delivery. As an anti‐tumor treatment, TAMs reprogramming molecules can be “loaded” inside or on the surface of the sEV to achieve their controlled and efficient delivery to the tumor niche. These loaded sEV will act as re‐educators of TAMs from their pro‐tumoral phenotype (M2‐like) toward an anti‐tumor phenotype (M1‐like). There are various techniques to enrich or load exogenous molecules to sEV, classified into two main types: pre‐isolation and post‐isolation techniques. In the former, the modifications are carried out at the level of the parental cell, which secretes sEV with the modifications designed. While in the second, the modifications are made directly on the sEV, after their isolation and purification (created with BioRender.com)

Among the physical procedures described to load the sEV are electroporation, sonication, surfactant treatment, freeze–thaw treatment, extrusion, and dialysis. Electroporation, sonication, and surfactant treatment generate pores in the phospholipid bilayer of sEV, which allows the incorporation of exogenous cargo. The freeze–thaw treatment, extrusion, and dialysis allow enriching the cargo during the membrane recombination processes. These methods have a variable loading efficiency that must be verified and ideally quantified, with moderate alterations in the integrity of the membrane that must be corroborated before starting any preclinical study. Electroporation is a physical technique that uses an electric field in the sEV resuspended in a conductive solution to generate temporary micropores, allowing the membrane's permeability and the diffusion of the exogenous cargo into the sEV. Using this technique, the sEV have been successfully loaded with drugs as doxorubicin,^126^ 5‐fluorouracil,^127^ and paclitaxel^128^; nucleic acids^17^, ^22^, ^104^, ^105^, ^107^, ^108^, ^109^, ^127^, ^129^, ^130^; proteins^131^; and theranostic nanoparticles for cancer.^132^ Sonication is a procedure that applies a mechanical shear force to weaken the integrity of the sEV membrane, which promotes the diffusion of exogenous molecules into the cargo of the nanovesicles. Such is the case of small molecules such as gemcitabine,^133^ doxorubicin,^134^ and paclitaxel^128^, ^134^, ^135^; proteins as catalase^136^; and various nanomaterials as gold nanoparticles.^136^ Surfactant treatment using, for example, saponin or triton allows to increase of the permeability of the sEV’ membrane through the formation of complexes between the surfactant molecules and the cholesterol of the membrane. Using this procedure, proteins have been successfully loaded.^131^, ^136^ The freeze–thaw strategy is a simple way to encapsulate into sEV various types of molecules such as drugs, proteins, and peptides.^136^ Through repetitive freeze–thaw cycles, it is also possible to fuse the liposome membranes (which carry the modification of interest) with the sEV membrane to develop a hybrid sEV.^137^ Extrusion is a process that mixes the sEV with the exogenous cargo in a lipid extruder, physically inducing the rupture of the sEV membrane and its subsequent mixing with the exogenous cargo as proteins.^131^, ^136^ Finally, dialysis allows to enrich the cargo of sEV with a drug of interest by mixing them on dialysis membranes or tubes, and then dialyzing them with stirring to obtain drug‐loaded sEV.^113^ Using this methodology, nucleic acid, specifically miRNA, siRNA, and single‐stranded DNA (ssDNA) have been loaded.^138^ A summary of the pre‐ and post‐isolation loading techniques can be found in Figure 2.

If the idea is achieving a TAM repolarization as a therapeutic approach, the reader should consider regulatory aspects when choosing a technique. Avoid pre‐isolation techniques based on genetic modifications or chemical‐induced cell phenotypic changes to minimize regulatory pitfalls. The latter applies too in the case of post‐isolation techniques, where the technic to choose should not require chemical modifications to avoid undesired traces in the isolated sEV. In the case of performing TAM repolarization for research purposes, the limitations are related to the availability of reagents and equipment necessary to carry out the technique.

### 6.2 Selective targeting of TAMS using engineered sEV

In the same line, the surface membrane of sEV can be altered by direct conjugation or genetic modifications in the parental cell. For this, click chemistry or ligases have been used to directly attach molecules to the sEV surface through covalent bonds to provide them a desired functionality.^16^, ^139^ This method has allowed to attach peptides to the phospholipid membrane to target specific anatomical sites.^140^, ^141^ Targeting M2‐like receptors—like CD206—in TAMs is the current most common strategy studied to increase therapeutic nanoparticle delivery. Using peptides like “UNO”^71^ (sequence: CSPGAK) or M2pep,^142^ different laboratories have successfully increased the internalization in vivo by TAMs of these conjugated nanoparticles. It is currently unknown if the modification of sEV with these peptides would generate similar biodistribution results.

Despite this, targeting strategies based only on M2‐like surface receptors do not guarantee that other subpopulations of macrophages expressing these same receptors generates “on‐target, off‐tumor” interactions and associated toxicity. Thus, an sEV delivery system for TAM's re‐education should also focus on tumor‐specific properties. For example, taking advantage of the acidic conditions (pH ~6.5) of the TME in several cancer models, pH‐sensitive compounds could be attached to the sEV surface, generating a controlled intake. Carboxymethyl chitosan (CMCS) is a biocompatible and biodegradable polysaccharide that alters its charge from predominantly negative to positive once it reaches the acidic conditions of the TME, increasing the interactions with cellular membranes. Using CMCS‐modified liposomes (CMCS‐SiSf‐CL), Yao et al. demonstrated an increased intake in a pH‐dependent manner in HCC cells, complemented with an enhanced in vivo tumoral accumulation.^143^

Another tumor‐specific property is the over‐expression of certain proteases like urokinase‐type plasminogen activator (uPA), membrane‐type serine protease 1 (MT‐SP1/matriptase), and legumain, which had been reported in several human tumors.^144^ To avoid the adverse effects associated with CAR‐T therapies, Han et al.^145^ designed a masked chimeric antigen receptor with a linker suitable to be cleaved by these proteases, resulting in a fourfold increase in the binding to its objective antigen after a protease treatment in vitro, decreased lung tumor growth in vivo and overall increased survival rate compared to its controls. In the context of a TAM's re‐education therapy using sEV, a similar strategy applying the cleavable linker could be evaluated. Recently, the Prostaglandin F2 Receptor Negative Regulator (PTGFRN) was identified as a “scaffold” protein suitable for genetically engineering sEV with defined therapeutic properties.^146^ Also, self‐peptides had been attached to the sEV surface by direct conjugation that act as a “don't eat me” signal, improving systemic circulation rate and decreasing the internalization by phagocytic cells.^139^ A genetic construct based on PTGFRN linked with the self‐peptides or other “do not eat me” motifs by the cleavable linker suitable to be degraded by tumor over‐expressed proteases could decrease off‐tumor internalization of engineered therapeutic sEV.

## 7 PERSPECTIVES AND FINAL CONCLUSIONS

The use of sEV as a nanoparticle drug delivery system is in full swing of study, development, and validation in biomedicine. As a nanocarrier, its success is based on its attributes such as its stability, biocompatibility, permeability, low toxicity, and low immunogenicity. However, biodistribution and pharmacokinetic studies of sEV show that they have a short half‐life in the systemic circulation, accumulating mainly in organs associated with the mononuclear phagocyte system (liver, spleen, lung). Macrophages are primarily responsible for their clearance, being considered a barrier to the bioavailability of sEV and, consequently, the therapeutic molecules they transport. This natural “tropism” that exists between sEV‐macrophages, although it may represent a disadvantage in its bioavailability as a carrier, it also means a strategic advantage for those therapies that involve macrophages. Indeed, TAMs are not immune to this (favorable) interaction with sEV, which has shown that in the TME, these phagocytic cells have a preferential uptake.

**FIGURE 3:**
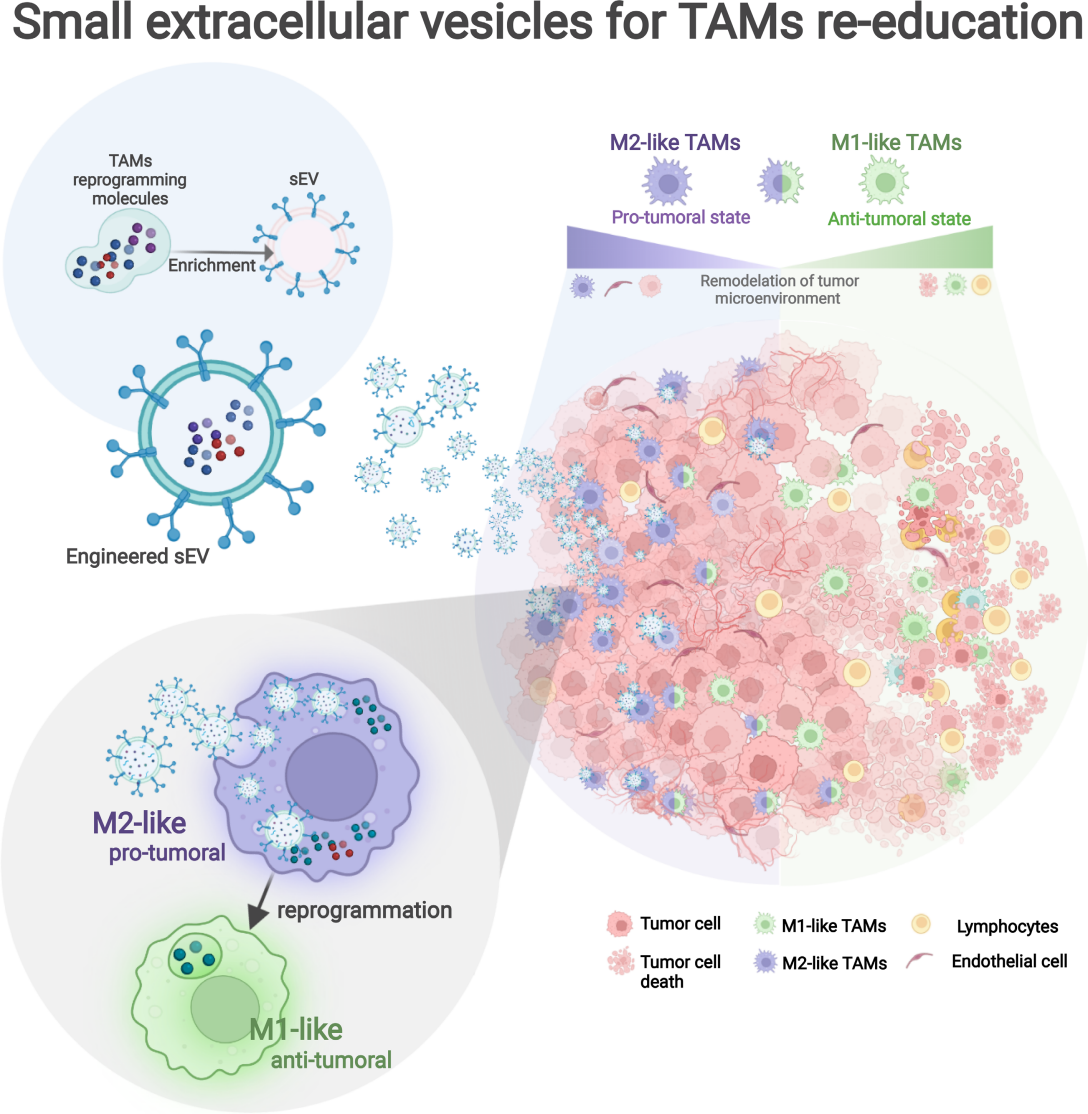
Small extracellular vesicles as therapeutic nanocarriers for the re‐education of TAMs. Small extracellular vesicles (sEV) possess several biological attributes as nanocarriers of exogenous molecules with therapeutic functions. Through different molecular techniques, it is possible to modify the native structure of sEV toward one designed to reprogram TAMs. The loading of specific molecules (drugs, proteins, nucleic acids, etc.) that generate TAMs reprogramming plus specific surface motifs that avoid off‐target interactions allow the development of an effective and safe anti‐tumor therapy. Taking advantage of the tropism of macrophages for sEV, the delivery of this therapeutic cargo can switch the pro‐tumoral and immunosuppressive properties of TAMs toward a pro‐inflammatory and anti‐tumoral phenotype. Balance modifications in the tumor microenvironment between pro‐tumoral and anti‐tumoral TAMs finally decrease tumor growth and increase leukocyte infiltration (created with BioRender.com)

In many solid tumors, TAMs are predominant populations of tumor‐infiltrating immune cells that correlate with a poor prognosis in patients. Although there are different subpopulations of TAMs with different transcriptomes and cell surface markers in the TME, pro‐tumoral M2‐like TAMs are the prominent phenotype present in tumors, which are responsible for stimulating various aspects of carcinogenesis and acts as a barrier to tumor sensitivity to immunotherapy. There is evidence that sEV‐based TAM reprogramming molecules nanosystem effectively reach their therapeutic target, triggering the reeducation of TAMs from their M2‐ to M1‐like state. Reprogramming TAMs toward an M1‐like antitumor phenotype limits tumor progression while enhancing antitumor immune responses (Figure 3). Undoubtedly, to minimize the potential side effects associated with off‐target interaction is essential that sEV‐based therapies should consider specific targeting on M2‐like TAMs without significantly affecting M2‐like macrophages resident in healthy tissues (located, for example, in the liver, spleen, or lung). Given the similarities between both cell populations, the nanovesicles drug delivery system should incorporate in their design modifications that allow increasing the control of activity in the TME, taking advantage of tumor‐specific properties such as acid pH or expression of specific proteases. Only in this way could one have control of the therapeutic precision.

Parallel to sEV design and engineering developments, it is imperative to advance in the productive scaling up and industrialization of clinical‐grade sEV production. The purpose is to expand and ensure the availability of therapy to all patients at the right time, either in monotherapy or in combination with other existing or emerging therapies. For this, it is essential to optimize the different stages of the production process to achieve greater efficiency and production performance of sEV, with high purity and maintaining its identity. Thus, for example, in upstream processing, the choice of cell culture system or culture medium can radically impact the performance of sEV production. Large‐scale cell culture systems, such as stacked multilayer devices for two‐dimensional (2D) culture; micro‐ or macro‐carrier‐based three‐dimensional (3D) cell culture systems; and hollow fiber bioreactor systems are recommended to advance in productive scaling; as well as the use of chemically defined culture media specifically designed to increase the secretion rate of sEV to the supernatant. In downstream processing, the single or combined use of methodologies such as size exclusion chromatography, ultrafiltration, and tangential flow filtration ensure an improvement in the yield and purity of the final preparation over the classical method based on ultracentrifugation. Likewise, lacking a single process that guarantees standardization between production batches, the determination of the sEV potency is a key tool to detect and resolve batch‐to‐batch inconsistencies and variations. Large‐scale production is necessary to ensure a continuous source and consistent isolation of sEV batches; therefore, large standardized quantities of sEV could be achieved by a combination of the different methods listed above and could be applied for large‐scale production of GMP‐grade and eventually clinical‐grade sEV, both of which are necessary to bring therapy closer to the patient.

## AUTHOR CONTRIBUTIONS

**Dario Donoso‐Meneses:** Conceptualization (equal); writing – original draft (equal); writing – review and editing (equal). **Aliosha Figueroa‐Valdés:** Conceptualization (equal); writing – original draft (equal); writing – review and editing (equal). **Nicolás Georges:** Conceptualization (equal); writing – original draft (equal); writing – review and editing (equal). **Hugo E. Tobar:** Conceptualization (equal); writing – original draft (equal); writing – review and editing (equal). **Francisca Alcayaga‐Miranda:** Conceptualization (equal); writing – original draft (equal); writing – review and editing (equal).

## CONFLICT OF INTEREST

Francisca Alcayaga‐Miranda received stipends from Cells for Cells. The other authors have no conflicts of interest to declare.

### PEER REVIEW

The peer review history for this article is available at https://publons.com/publon/10.1002/btm2.10349.

## ETHICS STATEMENT

This article does not contain any studies with human or animal subjects.

## Data Availability

The data that support the findings of this study are available from the corresponding author upon reasonable request.
